# Supplementary figures and images for: Cardiac Metastases in Neuroendocrine Neoplasms: A Single-Center Experience of Clinical Characteristics and Outcomes
Source: Cancers (Basel). 2025 Dec 6;17(24):3907. doi: 10.3390/cancers17243907 (PMC12730557; doi:10.3390/cancers17243907)

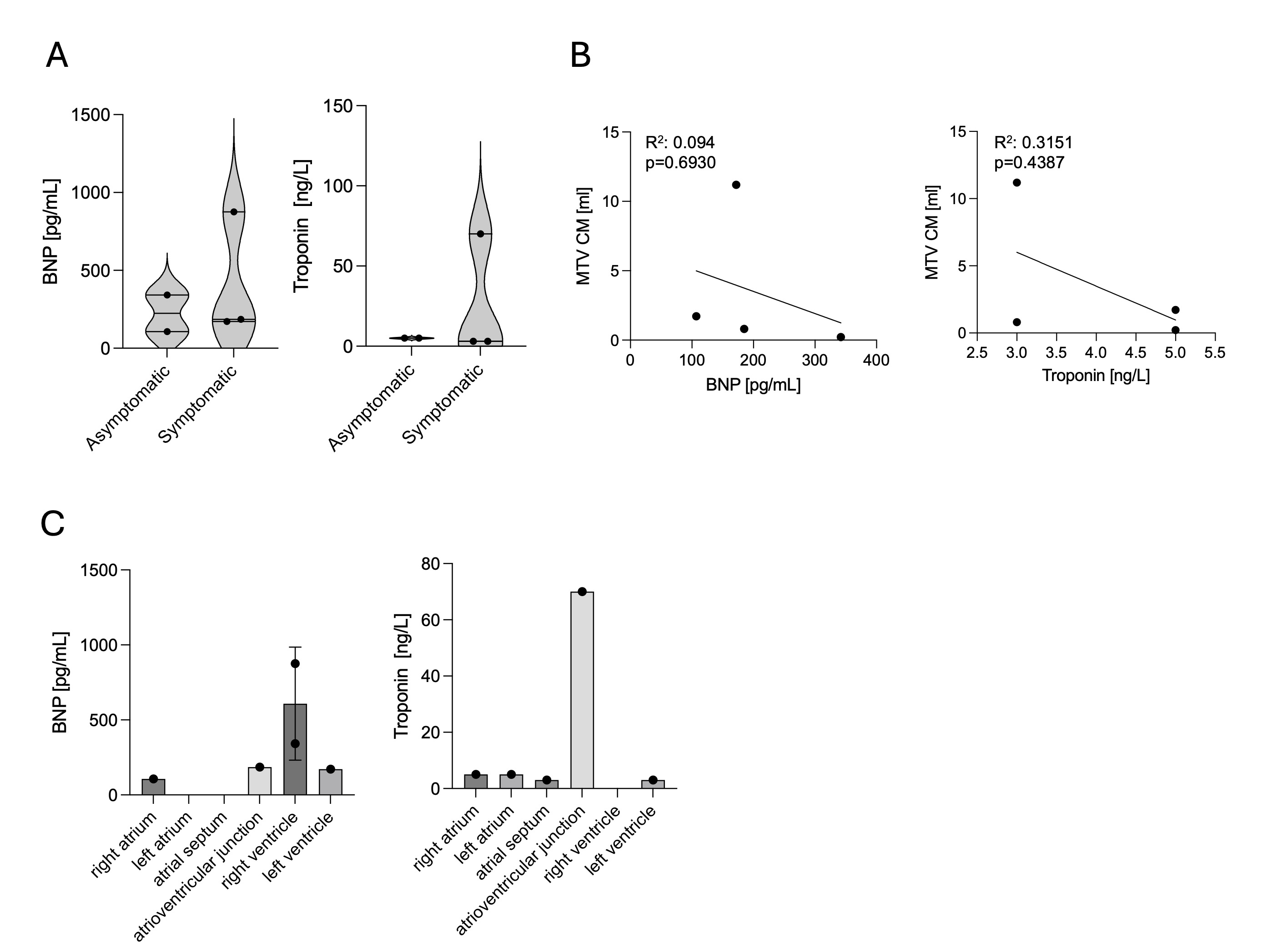

Supplement: Supplementary file 1 [file cancers-17-03907-s001.zip › PictureS1.jpg]
